# The Role of Tobacco Smoking in the Efficacy of Brief Alcohol Intervention: Results from a Randomized Controlled Trial

**DOI:** 10.3390/ijerph19105847

**Published:** 2022-05-11

**Authors:** Filipa Krolo, Sophie Baumann, Anika Tiede, Gallus Bischof, Kristian Krause, Christian Meyer, Ulrich John, Beate Gaertner, Jennis Freyer-Adam

**Affiliations:** 1 Institute for Medical Psychology, University Medicine Greifswald, Walther-Rathenau-Str. 48, D-17475 Greifswald, Germany; anika.tiede@med.uni-greifswald.de (A.T.); kristian.krause@stud.uni-greifswald.de (K.K.); jennis.freyer-adam@med.uni-greifswald.de (J.F.-A.); 2 German Centre for Cardiovascular Research, Partner Site Greifswald, Fleischmannstr. 42-44, D-17475 Greifswald, Germany; chmeyer@uni-greifswald.de (C.M.); ulrich.john@med.uni-greifswald.de (U.J.); 3 Department of Methods in Community Medicine, Institute of Community Medicine, University Medicine Greifswald, Walther-Rathenau-Str. 48, D-17475 Greifswald, Germany; sophie.baumann@med.uni-greifswald.de; 4 Department of Psychiatry and Psychotherapy, University of Lübeck, Ratzeburger Allee 160, D-23538 Lübeck, Germany; gallus.bischof@uksh.de; 5 Department of Prevention Research and Social Medicine, Institute of Community Medicine, University Medicine Greifswald, Walther-Rathenau-Str. 48, D-17475 Greifswald, Germany; 6 Department of Epidemiology and Health Monitoring, Robert Koch Institute Berlin, General-Pape-Str. 62-66, D-12101 Berlin, Germany; gaertnerb@rki.de

**Keywords:** brief intervention, alcohol, tobacco, efficacy, computer invention, counseling, moderator

## Abstract

This study investigated whether tobacco smoking affected outcomes of brief alcohol interventions (BAIs) in at-risk alcohol-drinking general hospital patients. Between 2011 and 2012 among patients aged 18–64 years, 961 patients were allocated to in-person counseling (PE), computer-based BAI containing computer-generated individual feedback letters (CO), and assessment only. PE and CO included contacts at baseline, 1, and 3 months. After 6, 12, 18, and 24 months, self-reported reduction of alcohol use per day was assessed as an outcome. By using latent growth curve models, self-reported smoking status, and number of cigarettes per day were tested as moderators. In PE and CO, alcohol use was reduced independently of smoking status (IRRs ≤ 0.61, *p*s < 0.005). At month 24, neither smoking status nor number of cigarettes per day moderated the efficacy of PE (IRR = 0.69, *p*s > 0.05) and CO (IRR = 0.85, *p*s > 0.05). Up to month 12, among persons smoking ≤ 19 cigarettes per day, the efficacy of CO increased with an increasing number of cigarettes (*p*s < 0.05). After 24 months, the efficacy of PE and CO that have been shown to reduce drinking did not differ by smoking status or number of cigarettes per day. Findings indicate that efficacy may differ by the number of cigarettes in the short term.

## 1. Introduction

Tobacco use and at-risk alcohol consumption are the most important modifiable risk factors for chronic disease development and death, accounting for 11.5% and 5.3% of all adult mortality, respectively [1,2]. The synergetic relationship between these health risk factors has been recognized in the past. Tobacco smoking may stimulate alcohol consumption and vice versa [3]. The joint effect of the substances can have additive or multiplicative effects on health [1,4]. Consumption of both substances can increase the risk of head and neck cancers by up to 35-fold higher than the use of one them [5]. In a general population sample in Germany, 55.1% of all adult at-risk drinkers also reported tobacco smoking, and 30.0% of tobacco smokers also drank alcohol in a risky manner [6].

Despite the strong association between alcohol use and tobacco smoking, the moderating effect of smoking on the outcome of BAI has not yet been investigated. Brief alcohol intervention (BAI) has been shown to be efficacious and effective by various systematic reviews and meta-analyses [7,8]. But tobacco smoking might reduce the efficacy as it can evoke alcohol drinking [9,10]. Namely, patients who smoked after treatment of their alcohol use disorder revealed earlier alcohol-related relapse than patients who did not smoke [11,12]. Further, a higher number of cigarettes per day at admission to alcohol treatment was associated with a higher increase in alcohol use at 6 months compared to a lower number of cigarettes per day [13]. Studies have shown that quitting smoking had a positive effect on the outcome of alcohol and drug treatments [14]. Alcohol consumers who stopped smoking were more likely to stop alcohol drinking compared to alcohol users who continued to smoke [15]. So far, studies on the influence of tobacco consumption on alcohol use are limited to alcohol treatment settings and heavy substance use [4,9,11,12,13,14]. To our knowledge, no study exists that investigated the moderating effect of smoking on the efficacy of BAIs in at-risk drinkers who are not alcohol dependent and who do not seek alcohol-specific treatment.

With regard to the increasing numbers of digital interventions, it also appears to be worthwhile to investigate whether certain subgroups benefit more so from in-person or computer-based interventions. In general hospital patients with at-risk alcohol use, we previously found that different levels of strain may play a crucial role. Whereas patients with lower alcohol use severity scores benefitted more so from computer-based than from in-person BAI, patients with higher scores tended to benefit more so from in-person than from computer-based BAI [16].

In order to improve efficacy of BAIs for all subgroups of target populations, the current study seeks to provide a first insight into tobacco smoking as a moderator of a BAI which has been delivered by a computer or in person. We hypothesized that, first, the efficacy of BAI is lower in patients who did smoke at first contact than in patients who did not. Second, we assumed that among smokers with a higher number of cigarettes per day BAI efficacy is lower than among smokers with a lower number of cigarettes per day. Moreover, we explored whether at-risk drinkers who also smoke or heavier smokers may benefit more so from one or the other BAI.

## 2. Materials and Methods

Data was derived from a three-arm randomized controlled trial “Testing delivery channels of individualized motivationally tailored alcohol interventions among general hospital patients: in-person v. computer-based, PECO” [17,18]. It is registered at ClinicalTrials.gov (NCT01291693). The ethics committee of the University Medicine Greifswald approved the study prior to data collection (BB07/10 and BB105/13). All trial participants provided informed written consent.

### 2.1. Participants

In 2011–2012, participants were recruited on 13 wards in four medical departments at the University Medicine Hospital Greifswald in Germany: internal medicine, general surgery, trauma surgery, and ear–nose–throat. Intensive care units were excluded. All consecutively admitted patients aged 18–64 years were approached by a research assistant and asked to respond to an electronic self-administrative lifestyle screening. Patients cognitively or physically incapable or terminally ill, patients with highly infectious diseases, patients discharged or transferred outside the study area within the first 24 h, patients previously recruited, patients with insufficient German language skills, and patients employed at the conducting research institute were excluded. Those study participants who screened positive for at-risk alcohol use according to national guidelines [19] were eligible for trial inclusion. The Alcohol Use Disorder Identification Test (AUDIT)–Consumption ≥4/5 [20,21] for women/men was used to assess at-risk alcohol use. Patients with an AUDIT score ≥ 20 [22,23] were excluded due to insufficient BAI efficacy in people with more severe alcohol problems [24]. Patients with no telephone were also excluded.

**Figure 1:**
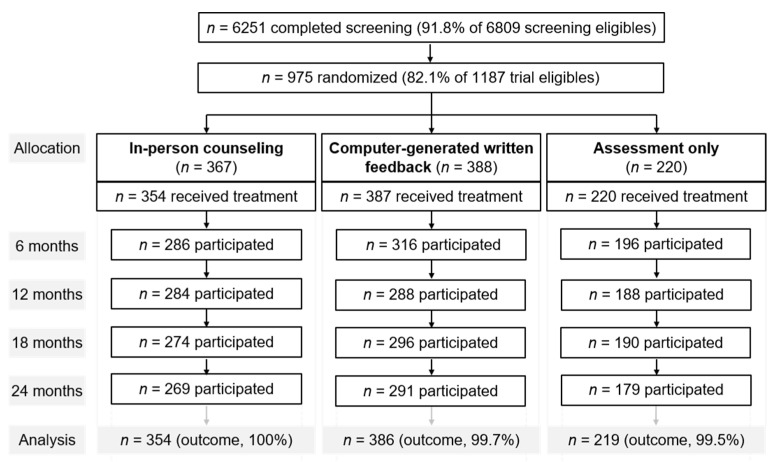
Participant flow by the study group.

Among all 10,593 patients aged 18–64 years who had been admitted to the participating wards, 6809 were eligible for screening, and 6251 (92%) completed the screening. Of the 1187 inpatients eligible for trial participation, 961 (81%) received their allocated intervention. Two participants, one with missing baseline covariate data and one with outlier data (342 g of pure alcohol per day), were excluded from analysis. The final sample included 959 patients with at-risk alcohol use (Figure 1). Participant flow according to CONSORT is reported in more detail elsewhere [18]. A sample of 975 patients with an allocation ratio of 2 in-person (PE):2 computer-based (CO):1 assessment only (AO) was calculated to be sufficient to detect small intervention effects concerning reduced alcohol use. To prevent study groups from exchanging intervention information, study group allocation rotated weekly over wards.

### 2.2. Interventions

As described elsewhere, PE and CO consisted of a similar content. They provided individually tailored feedback based on psychological behavior change theory and included three intervention contacts, normative feedback (i.e., on own data in comparison to others), and ipsative feedback (i.e., on own changes over time) [17,18]. Prior to each intervention, current alcohol use and the four dimensions of the transtheoretical model of behavior change [25] (i.e., stage of change, processes of change, decisional balance, self-efficacy) were assessed by using computer-based telephone interviews. According to the transtheoretical model, interventions are expected to be most effective when individually tailored to the person’s current motivational stage of change (precontemplation, contemplation, preparation, action, maintenance). As interventions are expected to achieve larger intervention effects, our interventions used all dimensions of the model for tailoring [26,27].

Both interventions were delivered at baseline on the ward (face-to-face) and after 1 and 3 months (by phone/mail). PE was primarily delivered by psychologists trained in motivational interviewing-based behavior change counseling [28]. Summed up across all three consultations, PE participants received 35 minutes of counseling (median), and 83% of the participants received two or three consultations. PE was delivered with acceptable adherence to motivational interviewing [18]. CO consisted of tailored 3–4-page computer-generated feedback letters, written in a patient-accepting, supportive, and non-confrontational style, and stage-matched manuals. Of the participants, 89% received two or three (of three) feedback letters. AO received minimal assessment at baseline only.

### 2.3. Follow-Up

Follow-up data was collected 6, 12, 18, and 24 months after recruitment by using computer-assisted telephone interviews (88%) or face-to-face interviews if no telephone contact could be realized. Incentives consisting of 5€ vouchers were paid before the follow-up at month 12 and after follow-up participation at months 6, 18, and 24. Overall, follow-up participation per time-point was 77–83%. The interviewers had not been informed about group allocation. Of the computer-assisted telephone interviews, 64% were conducted by student interviewers (97/47/49/60% at months 6/12/18/24) and 36% by research assistants who may have been involved in recruitment 1–2 years earlier.

### 2.4. Measures

#### 2.4.1. Outcome

Outcome was the average amount of pure alcohol used in grams per day determined by two questions concerning the month prior to assessment assessing frequency and quantity of drinking. Frequency was assessed by the question “In [month], how often did you have an alcoholic drink?” including five response categories: never (calculated as 0), once (calculated as 1), 2–4 times (calculated as 3), 2–3 times per week (calculated as 10), 4 times or more per week (calculated as 22). Quantity was assessed by the question “In [month], how many drinks did you typically have on a drinking day?” and the numbers of drinks containing beer (0.25 L)/wine or sparkling wine (0.125 L)/spirits (0.04 L) was asked for separately. To calculate grams per day, the number of drinks were multiplied with their according amount of pure alcohol (9.5 g/10.9 g/10.5 g) and summed up. A quantity-frequency product was determined, divided by 30.5, and rounded. 

#### 2.4.2. Smoking

Two indicators of current tobacco smoking at baseline were investigated as moderator variables: (1) Smoking status was assessed by the question “Are you a smoker currently?” including four response categories: “No, I have never smoked”, “No, I don’t smoke anymore”, “Yes, I smoke on a daily basis”, and “Yes, I smoke occasionally”. Current daily and occasional smokers were defined as current smokers and all others as current non-smokers. For descriptive purposes, participants who quit smoking were further asked if they quit smoking within the last six months or more than six months ago. (2) Among current smokers, heaviness of smoking was assessed by asking for the number of cigarettes smoked per day: “How many cigarettes/cigarillos/pipes/cigars do you usually consume on a day, when you smoke?”. To obtain the number of cigarettes per day in occasional smokers, they were further asked “On how many days per month do you smoke?”, and quantity and frequency were multiplied and divided by 30. Those who never smoked and former smokers were deemed as equivalent to 0 cigarettes per day. We chose few and short items because of the practicability in intervention studies in healthcare settings. At baseline, only 5.8% of all tobacco consumption was subject to cigarillos, pipes, and cigars. Thus, we did not differentiate among tobacco products. Regarding the number of cigarettes per day, applying M + 3SD, 17 outliers were identified, who smoked over 34 cigarettes per day at baseline. Thus, the highest number of cigarettes per day was set to 35.

#### 2.4.3. Covariates

Sociodemographics included sex (male/female), age (in years), having a partner (no/yes, including being married), employment status (employed/unemployed/other, e.g., pupil, student, or retiree), and education level (9 or less/10 to 11/12 or more years of school, including those still in school). The medical department to which the patients were admitted (internal medicine/surgical medicine/trauma surgery/ear–nose–throat) was recorded. Self-rated health was assessed by using a single item: “Would you say your health in general is: excellent (4), very good (3), good (2), fair (1), poor (0)?” [29]. Mental health was assessed by using the five-item mental health inventory [30,31] The score range was transformed to 0–100, with higher scores indicating better mental health. Alcohol-problem severity was assessed by using AUDIT [23]. It contains three items on current alcohol use and seven items on symptoms of alcohol-use disorders (range: 0 to 40, with higher scores indicating more severe alcohol problems). The stage in the motivation to change alcohol drinking (precontemplation, contemplation, preparation, action) was assessed by a 4-item staging algorithm, an adaption of measures previously introduced [32,33].

### 2.5. Data Analysis

To describe the development of alcohol use per day over the study period of 24 months, a latent growth curve model [34] (LGM) was used. LGMs allow one to model complex nonlinear growth curves over time, to reflect variance in growth curves, and to properly handle incomplete data [35]. In an LGM, repeated measures of the outcome are treated as indicators of latent growth variables that represent the outcome growth trajectory. In the current study, alcohol outcome data were highly positively skewed. Outcome variables were regressed on the latent growth variables by using a negative binomial model. Potential interactions between smoking and alcohol use were tested via likelihood ratio tests (LRTs). Functional form and variability of the growth curves were also tested via LRTs. As indicated by the later, the LGM included three latent growth variables representing the initial level of alcohol use (intercept), the linear rate of change (linear slope), and how much it was accelerating or decelerating over time (quadratic slope). The slope variances were fixed to 0 as the estimation of the models allowing the slope variances to be freely estimated was erroneous. To test if people with different smoking statuses benefitted differently from the computer-based and in-person interventions, smokers × study group and non-smokers × study group interactions were included in separate LGMs. For each follow-up timepoint and for each smoking status, net changes in alcohol use per day were calculated, defined as study group differences in the change from baseline to 6-, 12-, 18-, and 24-month follow-up, respectively. Net changes were given in incidence rate ratios (IRRs). The form of the interaction effects was further explored by the Johnson–Neyman technique plotting regions of significance and confidence bands for the intervention effect as a function of the continuous number of cigarettes [36]. *p* Values < 0.05 were considered statistically significant. Analyses were adjusted for all baseline covariates and conducted by using Mplus version 8.4 [37]. A maximum likelihood estimator with robust standard errors using numerical integration was chosen. Models were estimated under a missing at-random assumption [38] by using all available data. Multivariate logistic regression analyses including all covariates reported above revealed that dropout was not significantly associated with alcohol use, but predicted by fewer years of age and lesser school education, having no partner, poorer mental health, low motivation to change, and hospitalization on internal versus surgical medicine wards (*p*s < 0.05).

## 3. Results

### 3.1. Sample Characteristics

The final sample was composed of 718 men (74.9%) and 241 women (25.1%) with a mean age of 40.9 years (standard deviation [SD] = 14.1). Among the study participants, 190 (19.8%) had less than 10, 533 (55.5%) had 10–11, and 237 (24.7%) had 12 or more years of school education. Most of the participants were employed (*n* = 625, 65.1%) and in a partnership (*n* = 654, 68.1%). Participants reported drinking on average 15.2 g of pure alcohol per day (SD = 19.7). The mean AUDIT-C score was 6.0 (SD = 1.6); 83.6% of the participants scored 7 or less. As depicted in Table 1, the sample included 507 (52.9%) participants who smoked, 417 (43.5%) of which on a daily basis and 90 (9.4%) at least occasionally. The mean number of cigarettes per day was 7.4 (SD = 9.7). A more detailed description of the sample characteristics can be found elsewhere [18].

### 3.2. Moderator Smoking Status

In both intervention groups, smokers and non-smokers reduced alcohol use significantly at all time points (IRRs ≤ 0.61, *p*s < 0.05, Table 2). In AO, the smokers significantly reduced alcohol use up to month 18 (IRRs ≤ 0.61, *p*s 0.001), and the non-smokers significantly reduced alcohol use up to month 24 (IRRs ≤ 0.68, *p*s < 0.008).

In the LRTs, no improvement of model fit was achieved by adding interaction terms (*p*s > 0.05). LGMs were then used to explore potential tendencies of moderation. Concerning the differences between smokers and non-smokers, smoking status was not significantly associated with the efficacy of PE versus AO over all time points (IRRs 0.69 to 1.24, *p*s > 0.156, Table 3). Although non-smokers who received CO reduced alcohol use more strongly up to month 12 than non-smokers who received AO (IRRs 0.65 to 0.73, *p*s < 0.013), there were no significant differences in net changes between smokers and non-smokers (IRRs 0.85 to 1.15, *p*s > 0.438, Table 3). Smoking status was not significantly associated with the comparative efficacy of PE versus CO (IRRs 0.81 to 1.09, *p*s < 0.363, Table 3).

### 3.3. Moderator Cigarettes per Day

**Figure 2:**
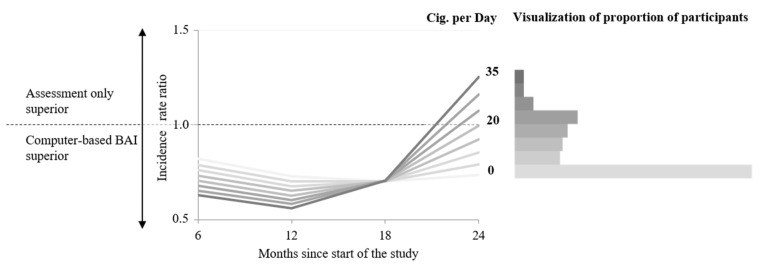
Effect of the computer-based BAI compared to assessment only.

**Figure 3:**
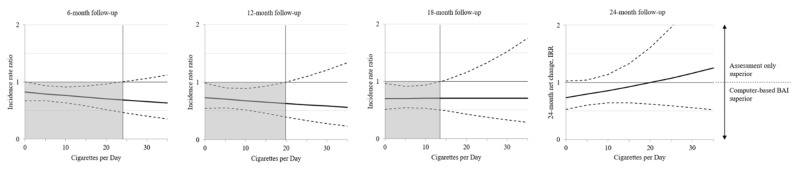
Plot of regions of significance for the effects of the computer-based BAI compared to assessment only.

LRTs showed no improvement of model fit by adding interaction terms (*p*s > 0.05). LGMs showed that over all time points, the effect of PE versus AO was the same regardless of the number of cigarettes per day. The Johnson–Neyman technique revealed no significance for any number of cigarettes after 24 months (*p*s > 0.05). Up to month 12, the effect of CO versus AO appears to be visually larger for persons smoking more cigarettes than for persons smoking fewer cigarettes (Figure 2). According to the Johnson–Neyman plot, statistical significance (intervention efficacy) was only found for persons smoking up to 24 cigarettes per day at month 6 and 19 cigarettes per day at month 12 (*p* < 0.05, Figure 3). At month 18, there seems to be no difference in the number of cigarettes regarding the effect of CO versus AO. Here, statistical significance was only found for persons smoking up to 14 cigarettes per day (*p* < 0.05, Figure 3). After our main time-point at 24 months, no significance and therefore no moderation effect can be found regarding the number of cigarettes per day (*p* > 0.05, Figure 3). Comparative efficacy of PE versus CO was not moderated by the number of cigarettes per day (*p*s > 0.05).

Effect of the computer-based BAI compared to assessment only on alcohol use per day in incident rate ratios (IRRs) among persons with different numbers of cigarettes per day. The horizontal dashed gray line (IRR = 1) indicates no difference between assessment only and computer-based BAI. Values below the dashed line (IRR ≤ 1) indicate superiority of the computer-based BAI; values above (IRR ≥ 1) indicate superiority of assessment only. The histogram shows the proportion of participants in each category of cigarettes per day.

The plot of regions of significance and 95% confidence bands for the 6-, 12-, 18-, and 24-month effects of the computer-based BAI compared to the assessment only on alcohol use per day as a function of numbers of cigarette per day. The effects (solid lines) are significant where the confidence bands (dashed lines) do not contain one (area shaded in gray).

## 4. Discussion

We investigated current tobacco smoking as a moderator of BAI efficacy, and of the comparative efficacy of in-person and computer-based BAI in particular. Three main findings are: first, neither smoking status nor heaviness of smoking moderated BAI efficacy after 24 months. Second, findings suggest that heaviness of smoking may moderate the efficacy of computer-based BAI in the short-term. Third, neither smoking status nor heaviness of smoking moderated whether patients benefitted more or less from one or the other ways of BAI delivery.

Although there is evidence that smoking may hinder effectiveness of alcohol treatment in treatment seeking alcohol-dependent patients, research on the influence of smoking in the context of BAIs for non-dependent persons with at-risk alcohol use only, has been scarce. In contrast to our hypotheses and studies based on alcohol-dependent and alcohol treatment-seeking patients [4,9,11,12,13,14], our findings suggest that smoking status and heaviness of smoking do not moderate BAI efficacy after two years. One possible explanation could be that smoking might include nicotine dependence and might be more severe in persons with alcohol dependence [39,40]. Higher consumption of one of the two substances results in higher consumption of the other [10,13,41], which means that moderator effects could be stronger in dependent persons. This might be a crucial difference to alcohol risk-drinking without dependence.

When looking at short-term follow-ups, it appears that heavy smokers might also benefit more from computer-based BAI (relative to assessment only) than light smokers. However, significance was only found for up to 19 cigarettes per day. The lack of significance for 20 cigarettes or more is likely due to reduced power to detect significant differences. Of the 959 participants in our sample, only 158 participants smoked 20 or more cigarettes per day. These short-term findings are rather in line with previous findings showing greater efficacy of these BAIs in patients with greater mental health problems [42].

Regarding the delivery mode of BAI, no differences were found between smokers and non-smokers and regarding heaviness of smoking. As found for smoking cessation interventions, different delivery modes worked equally well [43,44]. However, it may still be likely that the two ways of intervention delivery trigger different effects that may or may not lead to behavior change in one or the other subgroup. Future studies could investigate this more deeply.

For the purpose of screening and brief intervention in medical practice, our findings speak in favor of less interfering effects of the combination of tobacco smoking and alcohol risk-drinking than is the case among persons with alcohol dependence and tobacco smoking. It might be a future challenge to differentiate among these groups of recipients of brief interventions.

### Strengths and Limitations

This study has a few strengths. First, with 81% the reach of the target population was satisfactory. Each patient of the target population was approached and offered intervention participation, including those with low problem severity and those with no or only little motivation to change [45,46]. Second, several follow-ups over 24 months allowed a long-term view on the comparative efficacy of both types of intervention delivery for at-risk alcohol consumption using persons with or without tobacco smoking. Third, statistical methods that afford to properly handle missing values, skewed data, as well as the nonlinear relationship between outcome and time, were used. 

This study has a few limitations. First, analyses were based on self-reports, and underreporting of alcohol and tobacco consumption cannot be ruled out. However, by using biological data or external assessments other than self-report markers of alcohol or tobacco consumption it may be expected that the proportion of study participants among all eligible persons will be severely reduced. Alcohol and tobacco self-reports have been revealed by data to offer acceptable validity [47]. Second, the investigation was of exploratory nature. Although large and adequately powered as a whole, the sample was not powered to detect significant differences between smaller subgroups. However, from a public health perspective, our study revealed important new insights for the majority of smokers, namely for those with less than 20 cigarettes a day and with sufficient power to detect differences (*n* = 801, 83.52%). For the minority of smokers with 20 or more cigarettes per day, differences may be underestimated given the smaller number of participants and potential lack of power (*n* = 158, 16.48%). Future studies may be needed to replicate these findings. Third, changes in smoking over the timepoints, which may be likely after hospitalization, have not been considered. Related questions on whether changes in smoking mediate BAI effects or whether BAIs produce spill-over effects on smoking exceed the focus of this paper and should be investigated in the future. Fourth, the assessment of heaviness of smoking did not include the time to the first cigarette, the best single indicator for the assessment of nicotine dependence. However, as cigarettes per day has also proven to be reliable and valid [48], and to keep the assessment as brief and practicable as possible for interventions in healthcare settings, only the number of cigarettes smoked per day was assessed in our study. Finally, our sample is limited to proactively recruited at-risk drinking general hospital patients aged 18 to 64 years in one hospital in Germany. Replication is needed.

## 5. Conclusions

In the long-term, smoking status and heaviness of smoking did not moderate the efficacy of an effective in-person and computer-based BAI. However, in the short-term, heavier smokers may benefit more from computer-based BAI than lighter smokers. Further research should investigate the moderating effects of tobacco consumption on BAIs in adequately powered samples.

## Figures and Tables

**Table 1:** Moderator characteristics at baseline stratified by study group, *n* (%).

Moderators	In-Person (*n* = 354)	Computer-Based (*n* = 386)	Assessment Only (*n* = 219)
Current smoking status			
Non-smokers	166 (46.9)	179 (46.4)	107 (48.9)
Never smoked	76 (45.8)	70 (39.1)	38 (35.5)
Quit within the past 6 months	15 (9.0)	28 (15.6)	10 (9.4)
Quit more than 6 months ago	75 (45.2)	81 (45.3)	59 (55.1)
Smokers	188 (53.1)	207 (53.6)	112 (51.1)
Daily	158 (84.3)	172 (83.1)	87 (77.7)
Occasionally	30 (15.7)	35 (16.9)	25 (22.3)
Number of cigarettes per day 0	171 (48.3)	187 (48.5)	113 (51.6)
1–5	29 (8.2)	35 (9.1)	26 (11.9)
6–10	41 (11.6)	42 (10.9)	12 (5.5)
11–15	41 (11.6)	43 (11.1)	21 (9.6)
16–20	44 (12.4)	50 (13.0)	31 (14.2)
21–25	14 (3.9)	15 (3.9)	8 (3.7)
26–30	8 (2.3)	5 (1.3)	5 (2.3)
31–34	1 (0.3)	0 (0)	0 (0)
35	5 (1.4)	9 (2.3)	3 (1.4)
Mean number of cigarettes per day, (SD)	7.5 (9.5)	7.6 (9.8)	7.1 (9.9)

Note. *n* = number of cases, SD = standard deviation.

**Table 2:** Estimated changes in alcohol use from baseline to follow-up within study groups by smoking status.

	Month 6			Month 12			Month 18			Month 24		
Study Group	IRR	95% CI	*p*	IRR	95% CI	*p*	IRR	95% CI	*p*	IRR	95% CI	*p*
In-person BAI												
Smokers	0.61	0.48–0.76	<0.001	0.45	0.32–0.64	<0.001	0.42	0.29–0.60	<0.001	0.48	0.34–0.69	<0.001
Non-smokers	0.57	0.44–0.72	<0.001	0.41	0.28–0.59	<0.001	0.41	0.28–0.61	<0.001	0.58	0.40–0.85	0.005
Computer-based BAI												
Smokers	0.51	0.39–0.64	<0.001	0.36	0.25–0.52	<0.001	0.36	0.24–0.53	<0.001	0.49	0.33–0.73	<0.001
Non-smokers	0.49	0.38–0.64	<0.001	0.35	0.23–0.51	<0.001	0.34	0.23–0.52	<0.001	0.48	0.32–0.73	0.001
Assessment only												
Smokers	0.61	0.46–0.81	0.001	0.48	0.32–0.73	0.001	0.49	0.32–0.76	0.001	0.65	0.41–1.03	0.068
Non-smokers	0.68	0.51–0.90	0.008	0.54	0.35–0.83	0.005	0.50	0.32–0.78	0.003	0.54	0.35–0.83	0.005

Note. BAI = brief alcohol intervention, IRR = incidence rate ratio, CI = confidence interval, *p* = *p*-value.

**Table 3:** Incidence rate ratios (IRRs) of persons who smoke compared to persons who do not smoke with regard to study group net changes in alcohol use over 24 months.

	Month 6		Month 12		Month 18		Month 24		
Study Group	IRR	95% CI	IRR	95% CI	IRR	95% CI	IRR	95% CI	*p*
PE versus AO	1.24	0.35–1.70	1.24	0.78–2.00	1.02	0.65–1.67	0.69	0.40–1.13	0.156
CO versus AO	1.14	0.83–1.55	1.15	0.72–1.82	1.05	0.64–1.69	0.85	0.49–1.38	0.537
PE versus CO	1.09	0.82–1.45	1.08	0.72–1.67	0.98	0.64–1.55	0.81	0.52–1.29	0.363

Note. CO = computer-based intervention; PE = in-person intervention; AO = assessment only; CI = Confidence interval; *p* = *p*-value. Adjusted for sex, age, partnership, employment, school education, medical department, self-rated health, mental health, and motivational stage.

## Data Availability

Data available on request due to restrictions—The datasets generated and analyzed during the current study are not publicly available due to the German data protection law but are available from the corresponding author on reasonable request. Requests will be reviewed for reasonability and compliance with the study purpose and the participants’ informed consent.
